# Impact of prescription length supply policy on patient medication adherence in Thailand

**DOI:** 10.1186/s12913-023-09530-4

**Published:** 2023-05-24

**Authors:** Inthorn Jarujumrus, Suthira Taychakhoonavudh

**Affiliations:** 1 Pharmacy division, Phramongkutklao hospital, 315 Ratchawithi Rd., Thung Phaya Thai, Ratchathewi District, Bangkok, 10400 Thailand; 2 Department of Social and Administrative Pharmacy, Faculty of Pharmaceutical Sciences, Chulalongkorn University, Phayathai Rd, Wang Mai, Pathum Wan District, Bangkok, 10330 Thailand

**Keywords:** Adherence, Antihyperglycemic medications, Lipid-lowering medications, Difference-in-difference

## Abstract

**Background:**

Phramongkutklao Hospital is one of the largest military hospitals in Thailand. Beginning in 2016, an institutional policy was implemented in which medication prescription length was increased from 30 to 90 days. However, there have been no formal investigations into how this policy has impacted medication adherence among patients in hospitals. As such, this study evaluated how prescription length impacted medication adherence among dyslipidemia and type-2 diabetes patients who were treated at Phramongkutklao Hospital.

**Methods:**

This pre-post implementation study compared patients who received prescription lengths of 30 and 90 days based on information recorded in the hospital database between 2014 and 2017. Therein, we used the medication possession ratio (MPR) to estimate patient adherence. Focusing on patients with universal coverage insurance, we employed the difference-in-difference method to examine changes in adherence from before and after policy implementation, then conducted a logistic regression to test for associations between the predictors and adherence.

**Results:**

We analyzed data from a total of 2,046 patients, with equal amounts of 1,023 placed into the control group (no change to 90-day prescription length) and intervention group (change from 30 to 90-day prescription length). First, we found that increased prescription length was associated with 4% and 5% higher MPRs among dyslipidemia and diabetes patients in the intervention group, respectively. Second, we found that medication adherence was correlated with sex, comorbidities, history of hospitalization, and the number of prescribed medications.

**Conclusion:**

Increasing the prescription length from 30 to 90 days improved medication adherence in both the dyslipidemia and type-2 diabetes patients. This shows that the policy change was successful for patients in the hospital considered for this study.

**Supplementary Information:**

The online version contains supplementary material available at 10.1186/s12913-023-09530-4.

## Background

Over the last few decades, there has been an unprecedented rise in healthcare expenditures across the globe, [1, 2] with estimates projecting an increase of more than 200% from 2013 to 2040 [3]. Policymakers have thus employed a wide range of approaches to contain these climbing costs, including increased patient cost-sharing, switching from innovative brands to lower-priced generic alternatives, and reducing the length of time between prescription refills [1, 4, 5]. Although many such efforts have been effective, [6] they have also had unexpected spillover impacts on patients, thus increasing hospitalization and mortality rates while decreasing medication adherence [6, 7].

In 2016, Phramongkutklao Hospital implemented a measure known as the Extended Dispensing Policy (EDP), which aims to improve convenience and adherence among patients. Prior to implementation, patients with universal coverage (UC) were prescribed 30 days medication supplies, while those covered by the Civil Servant Medical Benefit (CSMB) scheme were given longer prescription lengths, specifically three-month treatments. Under the EDP, all patients with stable chronic diseases can now acquire up to three months of their prescribed medications, regardless of the provision type. While this allowance is expected to help patients, there is still a lack of evidence on the EDP’s specific impacts. Furthermore, the current situation of the national health policy of Thailand in 2023 which recommends the certain amount of prescription length supply is still unclear. As such, this study investigated how the 30-to-90-day increase in maximum prescription refill length has impacted medication adherence which is one of the key predictors of patient’s health outcome among patients with chronic disease who were treated at Phramongkutklao Hospital.

## Methods

### Data source

This study used hospital claims data from the Phramongkutklao Hospital Management System (PMKHMS) provided by Phramongkutklao Hospital, which is a quaternary care center and one of the largest military hospitals in Thailand. The database covered three main areas of necessary information, including demographic, clinical, and prescription data.

The demographic database covered a variety of patient characteristics, including sex, age, and the type of insurance coverage. Meanwhile, the clinical database contained details such as disease diagnostic data from the International Classification of Diseases, Tenth Revision (ICD-10), visit date, hospitalization date, and prescribed medications. Finally, the prescription database covered information on dispensed medications, dispensing date, dosing regimen, and medication quantities for each prescription.

### Study design and population

In this study, we employed a pre-post implementation study design to compare changes in medication adherence following EDP implementation.

**Fig. 1:**
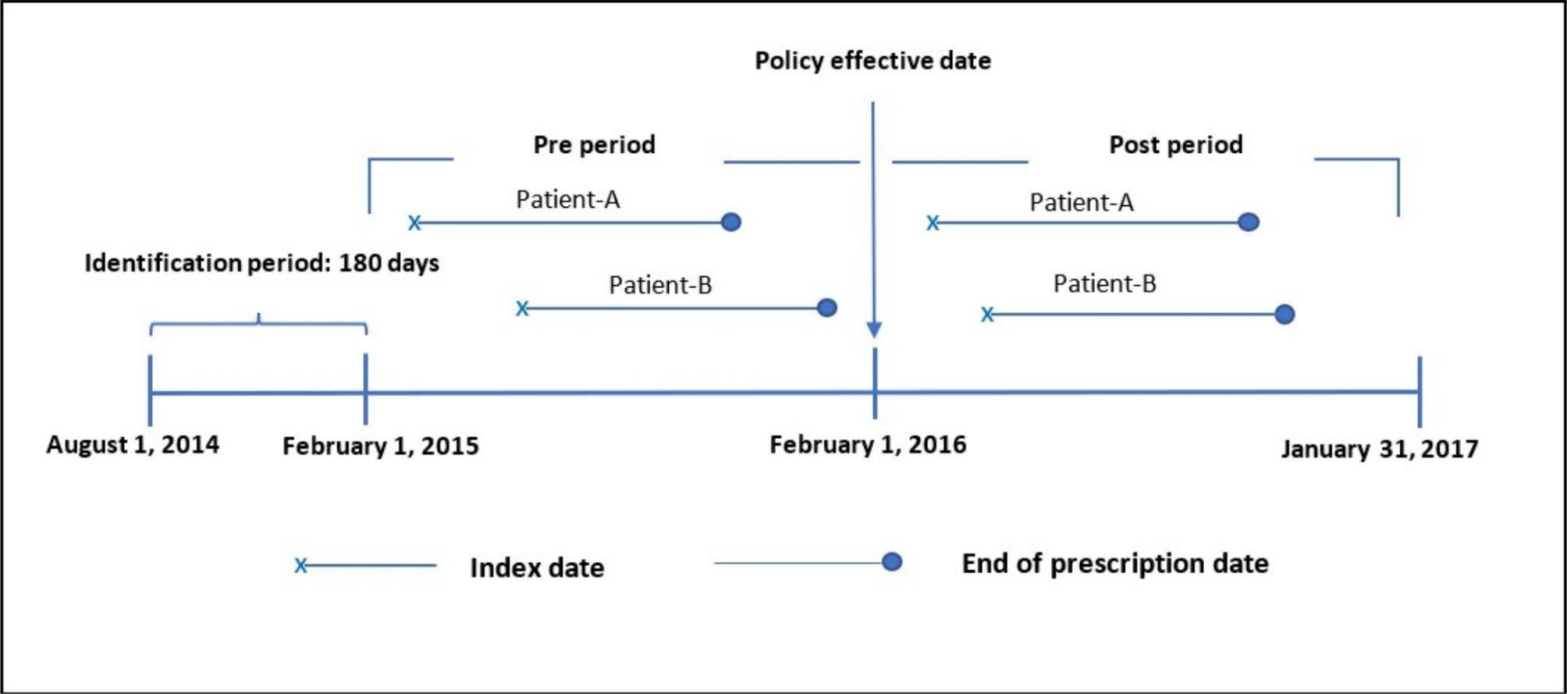
Study period and patient tracking showing the prescription refill pattern

As the EDP was enacted on February 1, 2016, our investigation covered both a 12-month pre-implementation period (February 1, 2015, through January 31, 2016) and 12-month post-implementation period (February 1, 2016, through January 31, 2017). For both periods, the first occurrences of dispensing were set as index dates. Patients included in this analysis had been on stable regimens for at least six months prior to the investigated period; that is, between August 1, 2014, and January 31, 2015, which was thus defined as the identification period. Patients were followed-up from the day of their index date until the day of their last dose of medication during the investigated period (Fig. 1).

#### Patient’s population and patient’s medical condition

Here, patients with UC were set as the intervention group, as they had experienced an increase in prescription length from 30 to 90 days, while patients under the CSMB scheme were set as the control group, as they were always allowed prescription lengths of 90 days.

We aimed an attention on the cardiovascular disease (CVD) which is the predominant cause of global death from non-communicable diseases (NCDs), especially ischemic heart disease and stroke [8]. Additionally, both of two diseases are correlated with alteration of lipid and glucose metabolism and primarily caused by dyslipidemia and diabetes mellitus (DM) [9, 10]. Moreover, we focused on patients with type 2 diabetes and dyslipidemia, since adherence to medication therapy is an important part of patient management in such cases. [11, 12]. Patients were diagnosed with diabetes mellitus and/or dyslipidemia using the ICD-10 diagnosis codes from their claims. For diabetes mellitus, we searched for codes E11, E13, and E14, which refer to non-insulin-dependent diabetes mellitus, other specific diabetes mellitus, and unspecified diabetes mellitus, respectively [13]. Meanwhile, patients with dyslipidemia were identified using code E78, which refers to disorders of lipoprotein metabolism and other types of lipidaemia. Based on recent clinical practice guidelines, we selected statins to represent medications for treating patients with dyslipidemia [14]. For patients with diabetes mellitus, we selected seven classes of antihyperglycemic medications, including sulfonylureas, non-sulfonylureas, biguanides, thiazolidinediones (TZDs), alpha-glucosidase inhibitors (AGIs), dipeptidyl peptidase-4 inhibitors (DPP-4 inhibitors), and sodium-glucose co-transporter-2 inhibitors (SGLT-2 inhibitors) [15].

#### Role of eligibility

Patients were subjected to the following inclusion criteria: (1) 18 years or older on their index date, (2) had at least one prescription in any class of antihyperglycemic medication or statins refilled between February 1, 2015, and January 31, 2016, and (3) had at least one prescription refilled from February 1, 2016, to February 1, 2017. By contrast, we excluded patients if they only received insulin prescriptions or glucagon-like peptide-1 receptor agonists (GLP-1 receptor agonists) for the treatment of diabetes mellitus at any time during the investigated period. Owing to the inadequacy of data on insulin dosing and GLP-1 receptor agonists, we could not accurately examine the amount of daily doses for injectable medication utilization [12].

#### Drug and dosage forms

Only adherence from tablets or capsules was assessed due to their dosage form can be measured accurately.

### Propensity score matching

The propensity scores were evaluated with a caliper of 0.001 via logistic regression using the following factors: age, sex, number of medications, Charlson Comorbidity Index (CCI) score, history of hospitalization, comorbidities, and class of medication. After generating weights, all patients in the intervention group were respectively matched with patients in the control group at a one-to-one ratio.

### Outcome variables

#### Medication adherence

This study used administrative prescription refills database; therefore, the medication possession ratio (MPR) was used as a proxy to verify medication adherence. MPR is a typical method, both in outcome research and standard procedure, for calculating adherence via claim databases [16, 17]. In this study, individual MPR assessments were computed from the database using the following formula:

1 $${\text{MPR = }}\frac{{{\text{Total days of medication provided}}}}{{{\text{Total days of follow - up}}}}$$

Medication adherence was assessed twice for each patient for both investigated periods; that is, pre- and post-EDP implementation with fixed duration of total days of follow-up. The pre-implementation period started from February 1st, 2015 through January 31st, 2016 (365 days), and the post-implementation started from February 1st, 2016 through January 31st, 2017 (366 days).

Computed MPR values were considered acceptable at ≥ 0.8, while values below 0.8 indicated nonadherence to medication [18, 19]. For patients receiving multiple therapies, MPR was calculated for each medication class, then a mean value was calculated [20, 21].

Patients who were prescribed with new medication or switch to other medication in the different medication class but still in the same therapeutic were identified as add-on and switch therapy, respectively. We assumed that patients who were switched their medication were stop taking their prior medication, then total days of follow-up were not fixed but calculated from the first date of their medication prescribed to the date of switching medication. Hospitalized patients were divided calculation into three parts which are before admission period, during admission period, and after discharged to the end of study period. Then average value of each medication class was assessed.

#### Independent variables and covariates

We adjusted for numerous confounding variables via regression model, including age, sex, number of medications prescribed, history of hospitalization, and CCI scores. We also incorporated time dummy variables to adjust for the time of the policy change, from 2015 to 2017.

### Data Analysis

To allow a baseline comparison, we conducted a chi-square test and *t-test* for the categorical and continuous variables, respectively. According to the natural experimental study design, we applied the difference-in-difference method. We identified an intervention and control group as well as a post-period and pre-period, then measured the differences based on the following two aspects: intervention/control group change and pre-/post-period change. We also conducted a multivariable logistic regression analysis using the data in both intervention and control groups to demonstrate the relationship between prescription length and medication adherence by adjusting for patient characteristics and disease-related variables.

### Sensitivity analysis

Owing to the lack of the standard value for MPR that used to identify medication adherence of the patients. We performed sensitivity analysis, applying various medication adherence thresholds of 0.50, 0.60, 0.70, and 0.90, included to pre-implementation and post-implementation in the study. Additionally, adjusted multivariable logistic regression was also conducted with these thresholds.

## Results

### Baseline characteristics

Initially, we obtained data on a total of 16,144 patients. Most descriptive characteristics showed differences between the intervention and control groups. After a propensity score adjustment, the final sample consisted of 2,046 patients, with 1,023 patients in each group. None of the demographic variables showed significant differences between these two final groups. An additional table file shows this in more detail [see Additional file 1].

### Change in medication adherence

Comparing the pre- and post-EDP periods, significant increases was identified in mean adherence (MPR) for patients with diabetes mellitus and dyslipidemia across the intervention and control groups. Looking only at those with diabetes mellitus, MPR increased by 14% and 9% in the intervention and control groups, respectively. Looking only at those with dyslipidemia, MPR increased by 22% and 18% the intervention and control groups, respectively. After propensity score matching, the tendency for pre-post changes in adherent patients for diabetes increased in both the control and intervention groups. For dyslipidemia, the intervention group reported a marked increase of 24.4%, while the control group showed a statistically significant increase of 23.1% (Table 1). Patients in the intervention group showed medication adherence nearly two times higher than those in the control group (adjusted OR than the patients in the control group) (Table 2). Women were less likely to adhere to their medications than men. Patients with histories of hospitalization during the study period were 14% less likely to adhere to their medications than those without such histories. The total number of prescribed medications was strongly correlated with medication adherence. Most comorbidities were significantly associated with medication adherence. Finally, CCI scores had positive effects on adherence.

**Table 1:** Changes in MPR for the intervention and control groups pre- and post-EDP

Measure	Unmatched		Propensity score matched	
**Type-2 diabetes patients**				
	Intervention	Control	Intervention	Control
No. of patients (n)	470	5463	400	400
MPR (mean ± SD)				
Pre-EDP	0.47 ± 0.27	0.53 ± 0.27	0.46 ± 0.26	0.49 ± 0.24
Post-EDP	0.58 ± 0.30	0.59 ± 0.28	0.60 ± 0.30	0.58 ± 0.28
Difference in mean (Post-Pre)	+ 0.11	+ 0.06	+ 0.14	+ 0.09
*P* value	< 0.001	< 0.001	< 0.001	< 0.001
Difference in Difference	+ 0.05		+ 0.05	
*P* value	< 0.001		< 0.001	
Adherence group (%, MPR ≥ 0.8)				
Pre-EDP	13.2%	17.8%	11.2%	10.5%
Post-EDP	26.0%	24.4%	28.0%	21.8%
Difference in %	+ 12.8%	+ 6.6%	+ 16.8%	+ 11.3%
*P* value	< 0.001	< 0.001	< 0.001	< 0.001
**Dyslipidemia patients**				
	Intervention	Control	Intervention	Control
Total (n)	1099	14,396	934	934
MPR (mean ± SD)				
Pre-EDP	0.46 ± 0.30	0.55 ± 0.32	0.44 ± 0.27	0.48 ± 0.28
Post-EDP	0.64 ± 0.32	0.68 ± 0.32	0.66 ± 0.32	0.66 ± 0.32
Difference in mean (Post-Pre)	+ 0.18	+ 0.13	+ 0.22	+ 0.18
*P* value	< 0.001	< 0.001	< 0.001	< 0.001
Difference in Difference	+ 0.05		+ 0.04	
*P* value	< 0.001		< 0.001	
Adherence group (%, MPR ≥ 0.8)				
Pre-EDP	17.8%	25.5%	13.9%	15.3%
Post-EDP	36.6%	41.0%	38.3%	38.4%
Difference in %	+ 18.8%	+ 15.5%	+ 24.4%	+ 23.1%
*P* value	< 0.001	< 0.001	< 0.001	< 0.001

EDP, Extended Dispensing Policy; MPR, medication possession ratio

**Table 2:** Logistic regression results showing EDP impacts on medication adherence

Variables	Adjusted odds ratio	95% CI	*P* value
Intervention x Period	1.94	1.80–2.09	< 0.001
Age in group (years)			
18–25	Reference		
26–50	0.58	0.45–0.74	< 0.001
51–75	0.88	0.69–1.12	0.305
>75	0.97	0.76–1.24	0.803
Gender			
Female	Reference		
Male	1.12	1.09–1.14	< 0.001
Number of medications prescribed at the index date			
>1	Reference		
1	3.20	3.12–3.29	< 0.001
Has history of hospital admission	0.86	0.84–0.89	< 0.001
Class of medications			
Sulfonylurea	0.19	0.18–0.19	< 0.001
Non-sulfonylurea	0.18	0.14–0.23	< 0.001
Metformin	0.50	0.49–0.52	< 0.001
Thiazolidinediones	0.21	0.19–0.22	< 0.001
Alpha-glucosidase inhibitors	0.36	0.34–0.39	< 0.001
DPP-4 inhibitors	0.42	0.39–0.44	< 0.001
SGLT-2 inhibitors	0.49	0.39–0.63	< 0.001
Statins	0.75	0.70–0.79	< 0.001
Comorbidities			
Acute myocardial infarction	1.14	1.06–1.21	< 0.001
Congestive heart failure	0.82	0.78–0.87	< 0.001
Cerebrovascular disease	0.86	0.83–0.89	< 0.001
Dementia	1.22	1.14–1.32	< 0.001
Chronic pulmonary disease	0.87	0.82–0.92	< 0.001
Peptic ulcer disease	1.19	1.05–1.36	0.006
Mild liver disease	0.87	0.82–0.92	< 0.001
Diabetes without chronic complication	1.42	1.36–1.48	< 0.001
Diabetes with chronic complication	1.72	1.66–1.79	< 0.001
Hemiplegia	0.65	0.52–0.83	< 0.001
Cancer	0.70	0.63–0.78	< 0.001
Solid tumor	1.44	1.06–1.95	0.02
AIDs	1.36	1.13–1.63	< 0.001
CCI score			
0	Reference		
1	1.18	1.15–1.22	< 0.001
>2	1.23	1.18–1.29	< 0.001

EDP, Extended Dispensing Policy; CCI, Charlson Comorbidity Index; AIDS, acquired immune deficiency syndrome

## Discussion

To the best of our knowledge, this was the first pre-post implementation study analysis to investigate the impacts of a policy designed to increase medication adherence based on administrative data from a quaternary care hospital in Thailand. Remarkably, the EDP provision was found to improve medication adherence in patients with dyslipidemia and type-2 diabetes, especially among those with UC insurance. Moreover, the EDP substantially increased the overall number of adherent patients, which is known to decrease healthcare costs and lower the risk of hospitalization [22–24]. These findings suggest that increasing prescription lengths from 30 to 90 days helps patients concentrate on their affiliated hospitals, thus producing continuity of care. In turn, healthcare professionals have more time to routinely take care of their patients.

Our findings are similar to those from previous studies showing increased medication adherence following measures to increase daily supplies [25–27]. Specifically, researchers found higher adherence due to increased prescription sizes for patients undergoing statin therapy, in which the refill period was raised from 30 to 60 days [25]. Further, a retrospective study showed that patients who received medication in four groups (antihypertensive, statins, oral hypoglycemic, and selective serotonin reuptake inhibitors) were more likely to adhere when given 90-day (vs. 30-day) prescription supplies [26]. In similar research, a study that extended the prescription length of statin medication from 30 days to 60 days or 90 days found correlations with increased medication adherence [27].

An inadequate baseline adherence rate was also noticed for diabetes and dyslipidemia patients, at only 11.2% and 13.9% for the one-year investigated period in the intervention group, respectively. This was mainly due to the limited prescription day supply up to 30 days before the policy was implemented. However, the proportions of adherent patients increased to 28.0% and 38.3% after the EDP was enacted, respectively. This was comparable to the overall adherence rate of 42.4% found in a study among patients who were prescribed antihyperglycemic medications [28]. Further, the rate of adherence found in this study was similar those reported in other studies that investigated medical and pharmacy claims databases [29, 30].

Although the clinical outcomes, such as HbA1c level, fasting plasma glucose (FPG), and lipid profiles, were not determined in this study, the literature reported their association with medication adherence. Andrew et al. [12] described that patients who do not adhere to oral glucose-lowering medications would have lower HbA1c decrease compared with adherence patients. Iloh et al. [31] explained that patients who adhere to their treatment were associated with plasma glucose controlled. Additionally, if patient adherence level to antidiabetic medication increased by 10%, level of HbA1c reduced by 0.16% as reported by Schectman [32]. Moreover, So-yeon et al. [33] concluded that adherent patients not only associated with better HbA1c level but also reduced fasting plasma glucose. For patients with dyslipidemia, increased length of statins from 30-day to 60-day at each prescription refill leads to better medication adherence and improved effectiveness of medication [25]. Another study that investigated the effects of expanding prescription length of statin from 30-day to 60-day and from 30-day to 90-day contributed to improvement in cholesterol level [27].

### Factor-affecting MPR

#### Patient-related factors

As for other factors, we observed a non-significant relationship between patient age and medication adherence. Specifically, patients aged 26 to 50 years were less likely to adhere to their medications than those aged 18 to 25. While older groups were more likely to be adherent, this trend was not significant. These findings support a previous systematic review that demonstrated an inverted U-shaped association between patient age and medication adherence [34]. In this study, we also found that being male was related to medication adherence, but a previous systematic review found inconsistent results on the relationship between this factor and the participants’ sex [35].

#### Therapy-related factors

There were also interesting findings on the quantity and type of medication. Here, patients who were prescribed only one medication were 3.20 times more likely to present adherence than those who were prescribed more than one. By contrast, some studies have shown that the number of prescription medications is not associated with medication adherence, [36] although there is evidence that the frequency of drug administration imposes an influence [37]. In this latter regard, a meta-regression analysis reported that patients who took cardiovascular disease drugs once daily were significantly more adherent than patients who took medications twice daily [38]. In this way, adjusting the frequency and quantity of medication could substantially promote adherence. We also found that all medication classes (especially AGIs, SGLT-2 inhibitors, biguanides, and statins) largely influenced lower medication adherence. This may be due to unique mechanisms of action. For example, AGIs that prevent digestion and delay the absorption of carbohydrates through the intestine may produce side effects such as flatulence, nausea, and diarrhea [39, 40]. Further, the remarkable glycosuric effect of SGLT-2 inhibitors promotes the excretion of glucose via urine, meaning that patients may experience frequent urination, dry mouth, and urinary tract infections [41, 42]. Moreover, common adverse effects are present when taking metformin, including abdominal bloating, vomiting, and metallic taste, [43] while statins may result in myalgia, abdominal discomfort, and joint pain [44]. Our findings support Bubalo et al., who found that patients were less likely to comply with therapy plans when there were frequent adverse events [45]. Therefore, to promote medication adherence, healthcare providers are not only responsible for counseling about the advantages of using the medication, but also providing appropriate recommendations through adverse events.

#### Disease-related factors

We also found that comorbidities were significantly associated with medication adherence. Patients diagnosed with congestive heart failure, cerebrovascular disease, chronic pulmonary disease, mild liver disease, hemiplegia, and cancer were less likely to adhere, while those with other diseases were more likely to adhere. In this regard, the literature offers some conflicting findings, with some studies showing higher adherence levels as the number of comorbid conditions increases, [46, 47] and others reporting lower medication adherence with complex comorbid conditions [48, 49]. There may be several explanations for these discrepancies. First, patients with many comorbidities may require complicated medical procedures. This may negatively affect how patients perceive their care plans, thus decreasing medication adherence [50]. Moreover, complicated treatments are highly associated with an accumulating number of medications, which may lead to omissions and reduced adherence [38, 51]. Adherence to therapy regimens also decreases substantially with time, especially in cases where medications are used to treat long-term chronic diseases [52]. This generally occurs when patients lack symptoms; for example, hypertension treatments may be associated with unfavorable adherence. As comorbidities and other factors may substantially influence medication adherence, it is important for health care providers to consider specific factors pertaining to each case, which entails targeted educational provisions and patient-centered care [29].

Our findings suggest that increased prescription lengths may enhance and maintain medication adherence for patients with chronic diseases, especially type-2 diabetes mellitus and dyslipidemia. This may point to the need for relevant policy changes at public hospitals in Thailand and elsewhere due to lack of up-to-date visible national policy direction. However, caution should be taken when generalizing these results, as they are based on a single-source database. Other important considerations when attempting to apply these results to other settings include the hospital type, patient demographics, medication class, and specific measure used to represent medication adherence.

### Limitations

It is worth noting that this study presents certain limitations. First, we examined administrative pharmacy claims from the Phramongkutklao Hospital database, which may not reflect the nature of all databases used in Thailand. This should be considered when comparing the results of policy changes in other hospitals. Second, the database was primarily operated for reimbursements, and did not include several predictors that have previously been associated with adherence, including education level, [53] race and ethnicity, [54] socioeconomic status, [55] adverse events with medications, [56] the relationship between healthcare providers and patients, [57] and social support [58]. Third, we used MPR as a proxy for medication adherence based on administrative prescription refills, but patients may not actually use the medications prescribed by their healthcare providers. Still, previous studies have shown that calculating adherence based on administrative data produces results that are consistent with those found in other approaches, including self-reported adherence, [59, 60] pill counts, [61] and direct measurements of serum drug concentrations [62]. In sum, this suggests that medication refill numbers reflect the amounts consumed by patients. Fourth, no data on any medications that patients may have acquired from other pharmacies or hospitals was considered in this study, which may have resulted in the underestimation of medication adherence. Finally, according to our study design was not randomized controlled, regression to mean (RTM) phenomenon was occurred and partially controlled. This determines the significance of further research to identify, reduce and handled RTM when possible [63].

## Conclusion

The EDP substantially promoted medication adherence in patients with dyslipidemia and type-2 diabetes. However, several other variables also affected adherence, some of which were related to specific therapies and the patients themselves. Efforts to determine clearly changeable variables that are correlated with both would benefit healthcare providers, patients, and national health services. Still, the current findings should aid in the development of new hospital policies, especially those aimed at ensuring that patients are consistent with their prescribed medications.

## Electronic supplementary material

Below is the link to the electronic supplementary material.

Supplementary Material 1

Supplementary Material 2

## Data Availability

The data that support the findings of this study are available from Phramongkutklao Hospital, but restrictions apply to the availability of these data, which were used under license for the current study, and so are not publicly available. Data are however available from the authors upon reasonable request and with permission of Phramongkutklao Hospital.

## Abbreviations

MPR: Medication Possession Ratio
EDP: Extended Dispensing Policy
UC: Universal Coverage
CSMB: Civil Servant Medical Benefit
PMKHMS: Phramongkutklao Hospital Management System
ICD-10: International Classification of Diseases, Tenth Revision
TZDs: Thiazolidinediones
AGIs: Alpha-Glucosidase Inhibitors
DPP4: Dipeptidyl Peptidase-4
SGLT2: Sodium-Glucose co-transporter-2
GLP-1: Glucagon-Like Peptide-1
CCI: Charlson Comorbidity Index
OR: Odds Ratio
