# Supplementary material for: Impact of prescription length supply policy on patient medication adherence in Thailand
Source: BMC Health Serv Res. 2023 May 24;23:533. doi: 10.1186/s12913-023-09530-4 (PMC10210410; doi:10.1186/s12913-023-09530-4)
Supplement: Supplementary file 1 — Supplementary Material 1 [file 12913_2023_9530_MOESM1_ESM.docx]

**Additional file 1: Comparison of patient baseline demographics between the intervention and control groups.**

| Baseline characteristics | Unmatched | | | Propensity score matched | | |
| --- | --- | --- | --- | --- | --- | --- |
|  | Intervention  (N = 1163) | Control  (N = 14981) | *P value* | Intervention  (N = 1023) | Control  (N = 1023) | *P value* |
| Age (years)  mean $\pm$ SD | 63.8 ± 13.5 | 65.6 ± 12.3 | < 0.01 | 64.2 ± 12.8 | 64.8 ± 12.2 | 0.3083 |
| Age group, n (%)  18-25 years  26-50 years  51-75 years  >76 years | 18 (1.6)  152 (13.1)  749 (64.4)  244 (20.9) | 11 (0.1)  1492 (10.0)  9846 (65.7)  3632 (24.2) | < 0.01 | 9 (0.9)  127 (12.4)  672 (65.7)  215 (21.0) | 2 (0.2)  119 (11.6)  683 (66.8)  219 (21.4) | 0.4604 |
| Sex, n (%)  Female  Male | 695 (59.7)  469 (40.3) | 7295 (48.7)  7693 (51.3) | < 0.01 | 609 (59.5)  414 (40.5) | 645 (63.0)  378 (37.0) | 0.112 |
| Number of medications per prescription at index date  Group, n (%)  Only one medication  More than one medication | 2.19 ± 1.36  455 (39.1)  708 (60.9) | 2.38 ± 1.62  5540 (63.0)  9441 (37.0) | < 0.01  0.147 | 2.2 ± 1.4  384 (37.5)  639 (62.5) | 2.3 ± 1.3  351 (34.3)  672 (65.7) | 0.2987  0.140 |
| History of admission, n (%)  Yes  No | 505 (43.4)  658 (56.6) | 3618 (24.1)  11363 (75.9) | < 0.01 | 409 (40.0)  614 (60.0) | 389 (38.0)  634 (62.0) | 0.389 |
| Morbidities, n (%)  Acute MI  CHF  Peripheral vascular disease  Cerebrovascular disease  Dementia  Chronic pulmonary disease  Rheumatic disease  Peptic ulcer disease  Mild liver disease  Diabetic without complication  Diabetic with chronic complication  Hemiplegia or paraplegia  Renal disease  Cancer  Moderate to severe liver disease  Metastasis solid tumor  AIDs | 51 (4.4)  69 (5.9)  15 (1.3)  144 (12.4)  12 (1.0)  46 (3.9)  57 (4.9)  3 (0.3)  45 (3.9)  465 (39.8)  115 (9.9)  2 (0.2)  187 (16.1)  27 (2.3)  1 (0.1)  3 (0.3)  5 (0.4) | 334 (2.2)  388 (2.6)  93 (0.6)  1403 (9.4)  327 (2.2)  555 (3.7)  247 (1.6)  96 (0.6)  548 (3.7)  4845 (32.3)  1180 (7.9)  26 (0.2)  1720 (11.5)  193 (1.3)  6 (0.04)  20 (0.1)  60 (0.4) | < 0.01  < 0.01  < 0.05  < 0.01  < 0.01  >0.05  < 0.01  > 0.05  > 0.05  < 0.01  < 0.05  < 0.01  < 0.01  < 0.01  >0.05  >0.05  >0.05 | 37 (3.6)  52 (5.1)  13 (1.3)  126 (12.3)  8 (0.8)  42 (4.1)  36 (3.5)  3 (0.3)  43 (4.2)  405 (39.6)  96 (9.4)  2 (0.2)  156 (15.2)  20 (2.0)  0 (0.0)  3 (0.3)  2 (0.2) | 32 (3.1)  46 (4.5)  6 (0.6)  130 (12.7)  10 (1.0)  34 (3.3)  32 (3.1)  2 (0.2)  32 (3.1)  430 (42.0)  116 (11.3)  1 (0.1)  150 (14.7)  25 (2.4)  0 (0.0)  1 (0.1)  0 (0.0) | 0.625  0.605  0.165  0.841  0.814  0.413  0.712  1.000  0.239  0.280  0.168  1.000  0.757  0.547  -  0.625  0.500 |
| CCI Score, Mean (SD)  Group, n (%)  0  1  2 | 1.3 ± 1.3  329 (28.3)  443 (38.1)  391 (33.6) | 0.9 ± 1.2  6395 (42.7)  5192 (34.7)  3394 (22.6) | <0.01 | 1.21 ± 1.21  301 (29.4)  408 (39.9)  314 (30.7) | 1.20 ± 1.20  315 (30.8)  390 (38.1)  318 (31.1) | 0.7252  0.7794 |
| Baseline medication used, n (%)  Sulfonylureas  Non-sulfonylureas  Biguanides  TZDs  Alpha-Glucosidase inhibitors  DPP-4 inhibitors  SGLT-2 inhibitors  Statins | 270 (14.6)  1 (0.1)  389 (21.1)  68 (3.7)  16 (0.9)  12 (0.7)  1 (0.1)  1089 (59.0) | 2998 (12.4)  53 (0.2)  4369 (18.0)  1216 (5.0)  530 (2.2)  779 (3.2)  55 (0.2)  14269 (58.8) | < 0.05  0.183  < 0.05  < 0.01  < 0.01  < 0.01  > 0.05  > 0.05 | 237 (23.2)  0 (0.0)  348 (34.0)  63 (6.2)  15 (1.5)  4 (0.4)  1 (0.1)  964 (94.2) | 266 (26.0)  1 (0.1)  383 (37.4)  60 (5.9)  7 (0.7)  5 (0.5)  0 (0.0)  978 (95.6) | 0.150  1.000  0.117  0.853  0.132  1.000  1.000  0.190 |

SD, standard deviation; Acute MI, acute myocardial infarction; CHF, congestive heart failure; AIDS, acquired immunodeficiency syndrome
