# Supplementary material for: Impact of prescription length supply policy on patient medication adherence in Thailand
Source: BMC Health Serv Res. 2023 May 24;23:533. doi: 10.1186/s12913-023-09530-4 (PMC10210410; doi:10.1186/s12913-023-09530-4)
Supplement: Supplementary file 2 — Supplementary Material 2 [file 12913_2023_9530_MOESM2_ESM.pdf]

Supplementary Information

Sensitivity Analysis

The results from sensitivity analysis in Supplementary Table 1,2 showed the same trend of pre-post changes of adherent in both type-2 diabetes and dyslipidemia patients with the higher threshold of medication adherence was set, the lesser patients can achieve the goal. For the adjusted logistic regression results displayed statistically significant with two thresholds of 0.5 and 0.6. These findings were exhibited in Supplementary Table 3.

Supplementary Table 1 Sensitivity analysis for MPR changed in type-2 diabetes patients using various threshold to determine adherence

Type-2 diabetes patients	Propensity score matched							
	MPR ≥ 0.5		MPR ≥ 0.6		MPR ≥ 0.7		MPR ≥ 0.9	
	Int (400)	Ctrl (400)	Int (400)	Ctrl (400)	Int (400)	Ctrl (400)	Int (400)	Ctrl (400)
Pre-EDP	38.8%	40.0%	28.8%	29.0%	17.5%	16.3%	11.2%	10.5%
Post-EDP	57.8%	56.8%	46.8%	44.5%	34.5%	29.5%	28.0%	21.8%
Difference in %	+ 19.0%	+ 16.8%	+ 18.0%	+ 15.5%	+ 17.0%	+ 13.2%	+ 16.8%	+ 11.3%
P value	< 0.001	< 0.001	< 0.001	< 0.001	< 0.001	< 0.001	< 0.001	< 0.001

Int, Intervention group; Ctrl, Control group

Supplementary Table 2 Sensitivity analysis for MPR changed in dyslipidemia patients using various threshold to determine adherence

Dyslipidemia patients	Propensity score matched							
	MPR ≥ 0.5		MPR ≥ 0.6		MPR ≥ 0.7		MPR ≥ 0.9	
	Int (934)	Ctrl (934)	Int (934)	Ctrl (934)	Int (934)	Ctrl (934)	Int (934)	Ctrl (934)
Pre-EDP	31.9%	36.0%	25.6%	27.9%	17.9%	20.0%	13.9%	15.3%
Post-EDP	60.4%	63.0%	52.8%	54.8%	44.9%	46.7%	38.3%	38.4%
Difference in %	+ 28.5%	+ 27.0%	+ 27.2%	+ 26.9%	+ 27.0%	+ 26.7%	+ 24.4%	+ 23.1%
P value	< 0.001	< 0.001	< 0.001	< 0.001	< 0.001	< 0.001	< 0.001	< 0.001

Int, Intervention group; Ctrl, Control group

Supplementary Table 3 Sensitivity analysis results showing EDP impacts on medication adherence.

Variable	Adherence threshold	Adjusted odds ratio	95% CI	*P* value
Intervention x Period	MPR ≥ 0.5	0.70	0.51-0.97	0.032
	MPR ≥ 0.6	0.69	0.51-0.95	0.023
	MPR ≥ 0.7	0.75	0.55-1.03	0.078
	MPR ≥ 0.9	0.89	0.64-1.24	0.483
